# The presence of circulating human apolipoprotein J reduces the occurrence of cerebral microbleeds in a transgenic mouse model with cerebral amyloid angiopathy

**DOI:** 10.1186/s13195-024-01541-5

**Published:** 2024-07-29

**Authors:** Anna Bonaterra-Pastra, Montse Solé, Silvia Lope-Piedrafita, Maria Lucas-Parra, Laura Castellote, Paula Marazuela, Olalla Pancorbo, David Rodríguez-Luna, Mar Hernández-Guillamon

**Affiliations:** 1 Neurovascular Research Laboratory, Vall d’Hebron Research Institute, Universitat Autònoma de Barcelona, Passeig Vall d’Hebron, 119-129, Mediterranean Building, 1st floor, lab 106, Barcelona, 08035 Spain; 2 Nuclear Magnetic Resonance Service, Universitat Autònoma de Barcelona, Cerdanyola del Vallès, Spain; 3 Department of Clinical Biochemistry, Clinical Laboratories, Vall d’Hebron University Hospital, Barcelona, Spain; 4 Stroke Research Group, Vall d’Hebron Research Institute, Barcelona, Spain; 5 Department of Bioquímica i Biologia Molecular i Institut de Neurociències, Universitat Autònoma de Barcelona, Bellaterra (Barcelona), Spain

**Keywords:** Amyloid-β, Apolipoprotein J, Cerebral amyloid Angiopathy, Cerebral microbleeds, MMP-12

## Abstract

**Background:**

Cerebral amyloid angiopathy (CAA) is characterized by amyloid-β (Aβ) deposition in cerebral vessels, leading to lobar cerebral microbleeds (CMB) and intracerebral hemorrhages (ICH). Apolipoprotein J (ApoJ) is a multifunctional chaperone related to Aβ aggregation and clearance. Our study investigated the vascular impact of chronic recombinant human Apolipoprotein J (rhApoJ) treatment in a transgenic mouse model of β-amyloidosis with prominent CAA.

**Methods:**

Twenty-month-old APP23 C57BL/6 mice received 25 doses of rhApoJ (1 mg/kg) (*n* = 9) or saline (*n* = 8) intraperitoneally for 13 weeks, while Wild-type (WT) mice received saline (*n* = 13). Postmortem brains underwent T2*-weighted magnetic resonance imaging (MRI) to detect hemorrhagic lesions. Aβ levels and distribution, cerebral fibrinogen leakage, brain smooth muscle actin (sma), and plasma matrix metalloproteinases and inflammatory markers were analyzed after treatments. Additionally, plasma samples from 22 patients with lobar ICH were examined to determine the clinical relevance of the preclinical findings.

**Results:**

rhApoJ-treated APP23 presented fewer cortical CMBs (50–300 μm diameter) (*p* = 0.012) and cortical larger hemorrhages (> 300 μm) (*p* = 0.002) than saline-treated mice, independently of Aβ brain levels. MRI-detected hemorrhagic lesions correlated with fibrinogen cerebral extravasation (*p* = 0.011). Additionally, rhApoJ-treated mice presented higher number of sma-positive vessels than saline-treated mice (*p* = 0.038). In rhApoJ-treated mice, human ApoJ was detected in plasma and in occasional leptomeningeal vessels, but not in the parenchyma, suggesting that its mechanism of action operates through the periphery. The administration of rhApoJ induced an increase in plasma Groα (*p* = 0.035) and MIP-1α (*p* = 0.035) levels, while lower MMP-12 (*p* = 0.046) levels, compared to the saline-treated group. In acute lobar ICH patients, MMP-12 plasma levels correlated with larger hemorrhage volume (*p* = 0.040) and irregular ICH shape (*p* = 0.036).

**Conclusions:**

Chronic rhApoJ treatment in aged APP23 mice ameliorated CAA-related neurovascular damage by reducing the occurrence of CMB. We propose that rhApoJ may prevent blood-brain barrier (BBB) leakage and CMB appearance partly through circulating MMP-12 modulation.

**Supplementary Information:**

The online version contains supplementary material available at 10.1186/s13195-024-01541-5.

## Background

Cerebral amyloid angiopathy (CAA) is caused by the cerebrovascular accumulation of amyloid, commonly amyloid-β (Aβ). Aβ is the product of the sequential processing of amyloid precursor protein (APP), resulting in peptides of different sizes prone to aggregate. The most common forms are Aβ_40_, which is mainly deposited in the brain vasculature, and Aβ_42_, which tends to deposit in the parenchyma forming the neuritic plaques, being a hallmark of Alzheimer’s disease (AD) [1]. Currently, there is no treatment for CAA, which is the main cause of lobar intracerebral hemorrhage (ICH) in adults > 55 years [2]. Furthermore, ICH recurrence is a common complication in CAA [2, 3]. ICH due to CAA typically affects lobar cerebral regions, whereas ICH due to chronic arterial hypertension, the other primary cause of ICH, usually affects deep cerebral regions [2]. Besides ICH, other clinical presentations of CAA include cognitive impairment, dementia and transient focal neurological episodes [4]. Aβ in blood vessels causes smooth muscle cell loss and vascular dysfunction, which could contribute to the appearance of cerebral microbleeds (CMB) [5]. Indeed, the presence of lobar CMBs is one of the main radiological and pathological features of CAA [4] and is associated with disease progression, recurring ICH [3], a higher likelihood of dementia [6] and severity of cognitive dysfunction [7]. Therefore, the occurrence of new lobar CMB could be considered a clinical endpoint to monitor future CAA treatments.

The prevalence of CAA pathology is particularly high in patients with AD [8]. Besides the biological overlap between AD and CAA based on Aβ metabolism and clearance, the interactions between them have several therapeutic implications. In particular, anti-Aβ passive immunotherapy, which is the approach that has been mostly advanced for its ability to reduce the parenchymal Aβ accumulation in early AD, has shown a high incidence of vascular complications detected by MRI, termed as amyloid-related imaging abnormalities (ARIA) [5]. ARIA can be classified as ARIA-E associated to vasogenic edemas, or ARIA-H characterized as cerebral microhemorrhages or hemosiderosis [9]. The pathophysiology of ARIA remains unclear, although several evidence suggest that the presence of CAA would be a risk factor for ARIA occurrence [5]. In this regard, recent case reports have shown fatal ICHs in patients who received monoclonal antibodies for AD with the subsequent confirmation of extensive CAA neuropathologic changes [10, 11]. Therefore, preventing the vascular symptoms associated with CAA pathology seems urgent to ensure the safety of this therapeutic strategy in AD patients.

Apolipoprotein J (ApoJ), also known as Clusterin (Clu), is a multifunctional chaperone that co-deposits with cerebrovascular Aβ in human brains [12–14] and can prevent Aβ fibrillogenesis [15, 16] and toxicity in vitro [17]. We previously reported that subchronic peripheral treatment with recombinant human ApoJ (rhApoJ), which represented a theoretical increase of 20% in the total plasma ApoJ, reduced cerebral Aβ levels and neurodegeneration in middle-aged (15-months-old) APP23 mice [18]. However, the impact of rhApoJ treatment on cerebrovasculature damage was not evaluated. APP23 is a mouse model of cerebral β–amyloidosis that presents parenchymal Aβ accumulation and prominent Aβ deposition in brain vessels, along with vascular dysfunction and spontaneous cortical and deep CMBs related to CAA in advanced ages [19–22]. In the present study we aimed to evaluate the cerebrovascular impact of increasing plasma ApoJ levels in aged APP23 mice by analyzing the occurrence of CMBs and the mechanisms triggered by the chronic treatment with rhAPoJ.

## Methodology

### Human recombinant ApoJ production and purification

The production of human recombinant ApoJ (rhApoJ) has been previously reported [18, 23, 24]. Briefly, transfected human embryonic kidney 293T cells (HEK293T) overexpressing rhApoJ were cultured in HYPERFlask systems (Corning Inc., Corning, NY, USA). The supernatants were used for protein purification with fast protein liquid chromatography (FLPC; AKTA Purifier 100 system, GE Healthcare Bio-Science Corp., Piscataway, NJ, USA) by Ni-affinity with HiScreen Ni FF columns (GE Healthcare) in the ICTS “NANOBIOSIS” Protein Production Platform Unit of the CIBER-BBN at the UAB, obtaining a purity > 75%^20^. The purified protein was dialyzed overnight (ON) against PBS in 10 kDa SnakeSkin Dialysis Tubing membranes at 4 °C (Thermo Fisher, Waltham, MA, USA). Protein concentration was quantified by bicinchoninic acid (BCA) assay (Thermo Fisher) and diluted to a final concentration of 300 µg of rhApoJ per PBS mL. Aliquots were stored at − 80 °C until use.

### In vivo **administration of rhApoJ**

APP23 C57BL/6 mice (B6.Cg-Tg (Thy1-APP) 3Somm/J) mice were obtained from The Jackson Laboratory (Bar Harbor, ME, USA) and C57BL/6 WT mice were obtained from Janvier Labs (Le Genest-Saint-Isle, France). Male APP23 mice were backcrossed with WT females, and the genotype was tested by Transnetyx (Cordova, TN, USA). The mice used were aged in a climate-controlled environment on a 12/12-hour light/dark cycle with food and water *ad libitum* in our animal facility. Two APP23 C57BL/6 mouse groups were treated for the experiments. Group 1 consisted of APP23 females 20 ± 0.5-months-old at the beginning of the experiment and received a chronic treatment of 25 doses of rhApoJ (1 mg/kg) (*n* = 9) or saline (*n* = 8) intraperitoneally two times per week during 13 weeks, whereas age-matched wild-type (WT) female mice received saline (*n* = 13). Sample size was calculated with ENE 2.0 software. Mice from Group 1 were euthanized 3 days after the last administration, after overnight (ON) fasting (Supplemental Fig. 1A). Group 2 was composed of 19.6 ± 2.3-months-old APP23 female mice that received one intraperitoneal dose of rhApoJ (1 mg/kg) (*n* = 4) or saline (*n* = 3) and age-matched WT female mice that received saline (*n* = 3). Mice from Group 2 were euthanized 30 min after administration (Supplemental Fig. 1B). Only females were employed to reduce variability, since they present more Aβ brain accumulation than males [19, 25]. All treated-mice were randomly allocated and administrations and analyses were performed in a blinded manner by an independent researcher. All the animals that survived were included in the study. Five animals died during the treatment (3 saline-treated WT and 2 rhApoJ-treated APP23), but neither mortality nor body weight loss was associated with the treatment or the genotype. All animal procedures were approved by the Ethics Committee for Animal Experimentation of Vall d’Hebron Research Institute and UAB (CEA-OH/10,888/1) and conducted from February to April 2020 in compliance with Spanish legislation (RD 53/2013) and European Union Directives (86/609/EEC). Experiments have been reported in compliance with the ARRIVE guidelines.

### Mouse cerebrospinal fluid (CSF), brain and blood collection

At the time of euthanasia, mice were anesthetized with isoflurane inhalation and CSF was sampled as previously described [18]. Briefly, CSF was collected by penetrating glass capillaries (0.5 mm in diameter) in the dura mater of the cistern Magna. Only clear CSF was considered for further experiments. After CSF extraction, blood was sampled via intracardiac puncture and centrifuged in ethylenediaminetetraacetic acid (EDTA)-tubes to collect plasma. Mice were then perfused with 20 mL of cold saline. For Group 1, this was followed by 20 mL of paraformaldehyde (PFA) 4%. Brains were rapidly removed, stored in 4% PFA at 4 °C for 72 h, and then transferred to PBS with 0.01% sodium azide for a minimum of 10 days for tissue stabilization. Brains from Group 2 were rapidly removed after saline perfusion. The left hemisphere was immediately immersed in 10% formalin for 72 h before paraffin embedding, whereas the right hemisphere was snap-frozen in liquid nitrogen.

### Plasma lipid profile

Levels of fasting triglycerides (TGs), total cholesterol (TChol) and high-density lipoprotein-cholesterol (HDL-C) in frozen plasma from Group 1 were automatically analyzed using an AI 5800 analyzer (Beckman Coulter, Pasadena, CA, USA) at Hospital Universitari Vall d’Hebron (HUVH) Clinical Laboratories (Barcelona, Spain). The quantification was carried out following the manufacturer’s instructions with reagents from Beckman Coulter. To determine the low-density lipoprotein-cholesterol (LDL-C) levels, HDL-C levels were subtracted from TC levels.

### Mouse ex vivo brain MRI

Mice from Group 1 underwent ex vivo T2*-weighted magnetic resonance imaging (MRI) in a horizontal magnetic system (7T, BioSpec 70/30USR, Bruker, Ettlingen, Germany) immersed in Galden^®^ D05 PFPE (Solvay, Bollate, Italy). MRIs were performed at the Nuclear Magnetic Resonance Service in Universitat Autònoma de Barcelona (UAB) using ParaVision 5.1 software (Bruker). T2*-weighted images were acquired using a fast-low angle shot sequence with the following parameters: repetition time, 700 ms; echo time, 8 ms; pulse angle, 40º; number of averages, 16; matrix, 160 × 160; field-of-view, 1.28 × 1.28 cm^2^; slice thickness, 0.3 mm; and 28 slices. The voxel size was 0.08 × 0.08 × 0.3 mm^3^, and the total acquisition time was 22 min. Spherical hypointense signals on T2* were visually counted as hemorrhagic lesions and classified as CMB (50–300 μm diameter) or larger hemorrhages (> 300 μm) [22]. To avoid counting the same hemorrhagic lesion multiple times, its presence was carefully controlled across consecutive slices. Hemorrhagic lesions were also classified according to their location as lobar (cortex) or deep (thalamus and basal ganglia). We have previously reported a correlation between hypointensities detected with this protocol and Prussian Blue staining, affirming ferric deposition and, consequently, indicating hemorrhagic lesions [19]. Hemorrhagic lesion volume was quantified using ImageJ Software (NIH, Maryland, MD, USA). Post-MRI, brains were immersed in 10% formalin for 12 h and paraffin-embedded.

### Mouse brain homogenates

The right brain hemispheres of Group 2 were homogenized in cold sucrose buffer (0.32 M sucrose (Sigma‒Aldrich, Saint Louis, MO, USA) and 5 mM HEPES (Thermo Fisher)) with a Dounce homogenizer. RIPAx2 lysis buffer containing protease and phosphatase inhibitors was added to the homogenates and then centrifuged for 15 min at 15,000 g at 4 °C to obtain the final supernatant as the brain homogenate.

### Thioflavin S (ThS) staining

ThS staining for fibrillary Aβ was performed on 4 μm-thick sagittal paraffin-embedded brain sections, which were obtained at an equivalent depth and always included the hippocampus and striatum. Sections were deparaffinized for 1 h at 60 °C and rehydrated before staining. Sections were then immersed in 1% ThS solution (Sigma‒Aldrich) dissolved in 75% ethanol for 30 s followed by 0.1% Ths for 1 min. Sections were then washed with 75% ethanol, dehydrated and mounted in DAPI-containing mounting media (Vector Laboratories, Burlingame, CA, USA) for nuclei counterstaining.

### Resorufin staining

Resorufin staining for specific vascular Aβ detection [26] was performed in 4 μm-thick sagittal brain paraffin-embedded sections, which were obtained at an equivalent depth and always included the hippocampus and striatum. Sections were deparaffinized for 1 h at 60 °C and rehydrated before staining. Sections were washed with PBS and permeabilized in PBS 0.2% Triton (PBST) before being immersed in a PBS solution with resorufin 1 mM (Sigma‒Aldrich) for 5 min. Finally, samples were rinsed with PBS, PBS-50% ethanol, dehydrated and mounted in DAPI-containing mounting media (Vector Laboratories).

### Prussian blue staining

Prussian Blue, which stains ferric iron and hemosiderin complexes, was performed to detect the presence of cerebral hemorrhagic lesions. The staining was performed in 4 μm-thick sagittal brain paraffin-embedded sections, which were obtained at an equivalent depth and always included the hippocampus and striatum. Sections were deparaffinized for 1 h at 60 °C and rehydrated before staining. Sections were stained with a commercial Prussian blue iron stain kit (Polysciences Inc, Warrington, PA, USA) following the manufacturer’s instructions. Briefly, sections were incubated with potassium ferrocyanide and hydrochloric acid solution (1:1) for 40 min at room temperature (RT). Sections were then rinsed in water and counterstained with Nuclear Fast Red for 5 min. Finally, sections were dehydrated and mounted with DPX (Sigma‒Aldrich).

### Immunohistochemistry

Immunohistochemical staining was performed to specifically detect Aβ_40_, Aβ_42_, sma and Cluster of Differentiation 68 (CD68). The staining was performed in 4 μm-thick sagittal brain paraffin-embedded sections, which were obtained at an equivalent depth and always including the hippocampus and striatum. Sections were deparaffinized for 1 h at 60 °C and rehydrated before staining. Sections were then incubated with citrate buffer (10 mM sodium citrate, 0.05% Tween 20, pH = 6) at 95 °C for 30 min for antigen retrieval. Blocking was performed with 1.5% glycine, 10% fetal bovine serum (FBS) and 0.2% TBS- 1% Tween 20 diluted in TBS for Aβ_40_ and Aβ_42_ detection, 10% goat serum and 0.1% Triton diluted in TBS for sma detection, and, 10% goat serum and 1% bovine serum albumin (BSA) in PBS- 0.1% Tween20 for CD68 detection for 1 h at RT. Sections were then incubated ON at 4 °C with the following primary antibodies diluted in blocking buffer: rabbit polyclonal anti-Aβ_40_ (1:5000, #AB5074P, Millipore, Temecula, CA, USA), rabbit polyclonal anti-Aβ_42_ (1:5000, #AB5078P, Millipore), rabbit polyclonal anti-sma (1:1000, # ab5694, Abcam, Cambridge, UK) and rabbit polyclonal anti-CD68 (1:5000, #ab125212, Abcam). Endogenous peroxidases were blocked with 1% hydrogen peroxide treatment for 15 min, and samples were then incubated with biotinylated anti-rabbit IgG diluted in blocking buffer (1:1000, Vector Laboratories) at RT for 1 h, followed by streptavidin-horseradish peroxidase (HRP; 1:1000, Vector Laboratories) 1-hour incubation at RT. Finally, the sections were treated with diaminobenzidine (DAB; Dako, Glostrup, Denmark) for 15 s, and Harris hematoxylin solution (Sigma‒Aldrich) was used for contrast staining. For mounting, sections were dehydrated and mounted with DPX (Sigma‒Aldrich). Sections incubated with only the secondary antibody were used as negative controls.

### Immunofluorescence

Immunofluorescence staining was used to specifically detect fibrinogen, ionized calcium-binding adapter molecule 1 (Iba1) and human ApoJ (hApoJ). Staining was performed in 4 μm-thick sagittal brain paraffin-embedded sections, which were obtained at an equivalent depth and always included the hippocampus and striatum. Sections were deparaffinized for 1 h at 60 °C and rehydrated before staining. Sections were then incubated with citrate buffer (10 mM sodium citrate, 0.05% Tween 20, pH = 6) for 30 min at 95 °C to enhance antigen exposure. Then, slices were blocked with 3% BSA in PBS-0.1% Tween 20 for fibrinogen and 10% FBS in PBS-0.1% Tween 20 for human Iba1 and hApoJ. For fibrinogen staining, sections were then incubated ON at 4 °C with the conjugated antibody fibrinogen-FITC (1:100, #F0111, Dako). To detect Iba1 and human ApoJ, sections were incubated ON at 4 °C with the corresponding primary antibody Iba1 (1:200, #ab178847, Abcam) and hApoJ (1:50, #ab39991, Abcam) and incubated with the secondary antibody anti-rabbit-IgG-488 for Iba1 (1:500, Invitrogen, Waltham, MA, USA) and anti-goat-IgG-488 for hApoJ (1:500, Invitrogen) diluted in blocking buffer for 1 h at RT the next day. Sections were finally mounted with DAPI counterstaining (Vector Laboratories). Sections incubated with only the secondary antibody were used as negative controls.

### Tomato-lectin staining

Tomato-lectin-594 staining was used to determine capillary density. To avoid interference from lectin-positive staining of microglia, stainings were performed in a brain region poorly affected by Aβ deposition, such as in the striatum, where microgliosis was not detected. Staining was performed in 4 μm-thick sagittal brain paraffin-embedded sections, which were obtained at an equivalent depth and always including the hippocampus and striatum. Sections were deparaffinized for 1 h at 60 °C and rehydrated before blocking with 1% BSA, 10% goat serum in PBS-0.1% Tween 20. Then, sections were incubated ON at 4 °C with tomato-lectin-594 (1:100, DL-1177, Vector Laboratories). Finally, sections were mounted with DAPI counterstaining (Vector Laboratories).

### Quantification after staining/ immunodetection of brain sections

Images from all histological sections were obtained with a 20x objective in the Panoramic 250 scanner (3DHistech, Budapest, Hungary) and then digitized and processed by Case Viewer Software (3DHistech). Quantifications were performed with ImageJ software (NIH). For all staining techniques, except resorufin, the brain region of interest was delimited (cortex, hippocampus, thalamus, or the whole brain except for the olfactory bulb and cerebellum) and the area was measured. The number of positive objects was identified by thresholding and divided by the area (# of deposits/area (mm^2^)). Additionally, the percentage of the positive area (%) and the average size of the deposits (mm^2^) were calculated. For the quantification of vessels positive for Aβ, they were manually determined in ThS-,

Resorufin-, Aβ_40_-IHC-stained and Aβ_42_-IHC-stained sections in the selected area (meninges, cortex, hippocampus, thalamus or the whole brain except for the olfactory bulb and cerebellum). Vessels positive for fibrinogen and sma were also manually determined in the whole brain, specifically in the meninges, cortex, hippocampus, thalamus and striatum. The number of capillaries stained by tomato-lectin was manually determined in three areas, each measuring 273.282 μm², and the density was expressed as the mean number of capillaries per unit area (mm²). Finally, to calculate the number of Aβ plaques, the number of positive vessels was subtracted from the number of positive objects identified by thresholding, both in ThS and Aβ_40_ staining, and divided by the area (# of plaques/area (mm^2^)).

### Human study population

The cohort was recruited in the Stroke Unit of HUVH and included 22 participants who suffered at least one acute ICH in a lobar location detected by computed tomography (CT) scan [27]. The ICH volume was CT-measured using semiautomatic Hounsfield-unit, threshold-based, computerized planimetry software [27]. The hemorrhage’s shape was analyzed according to Barras et al. [28] on a 1–5 scale, with 1 being the most regular shape and 5 the most irregular. Demographic characteristics, vascular risk factors, medication and ApoE genotype were recorded. Blood was collected in the acute phase (< 24 h from CT) in EDTA-containing Vacutainer tubes (Becton Dickinson, Franklin Lakes, NJ, USA) and centrifuged at 4 °C for 15 min at 2500 rpm. Clinical parameters in plasma were evaluated by HUVH laboratories. The study was approved by the local Clinical Investigation Ethical Committee (PR(AG)269/2017) and adhered to the Helsinki Declaration.

### Single and multiplexed ELISAs

Aβ_40_ quantification in mice’s CSF was performed by a commercial enzyme-linked immunosorbent assay (ELISA) kit (#KHB3481, Invitrogen). Plasma and brain levels of human ApoJ were analyzed with an ELISA kit specific for human ApoJ (#3713-1HP-1, Mabtech, Stockholm, Sweden). The levels of plasma inflammation markers (Groα, Il-1β, IL-17 A, MCP-1, MIP-1α, MIP-1β, MIP-2α) and matrix metalloproteinase (MMP) (MMP-2, MMP-3, MMP-8, proMMP-9, MMP-12) were evaluated by multiplexed ELISAs (#EPX080-20832-901, Thermo Fisher; #MMMP1MAG-79 K and # MMMP2MAG-79 K, Merck, Darmstadt, Germany). MMP-12 levels in human plasma were evaluated by an ELISA kit (#EH327RB, Thermo Fisher). Extreme outlier values detected by SPSS software were discarded from the analysis.

### Statistical analysis

Linear variable distributions were assessed using the Shapiro‒Wilk test. Normal distributions were subjected to a t-test or one-way ANOVA with Bonferroni correction. For non-normal data, a Mann‒ Whitney test or Kruskal‒Wallis test with Dunn’s post hoc test for multiple comparisons was performed. Normal variables are expressed as the mean ± SD, whereas non-normal variables are expressed as the median [interquartile range]. Associations between categorical variables were examined using crosstab and chi-squared or Fisher tests as appropriate. For correlations, the Spearman coefficient Rho was used. To adjust MMP-12 plasma level associations in the ICH cohort, backward linear regressions were performed, estimating the slope (B) with a 95% confidence interval (CI) for each case. Statistical analyses were conducted using SPSS Statistics 21 (IBM Corporation, Armonk, NY, USA) and GraphPad Prism 9 (Prism, Pleasant Hill, CA, USA). A *p* value below 0.05 was considered statistically significant.

## Results

**Fig. 1:**
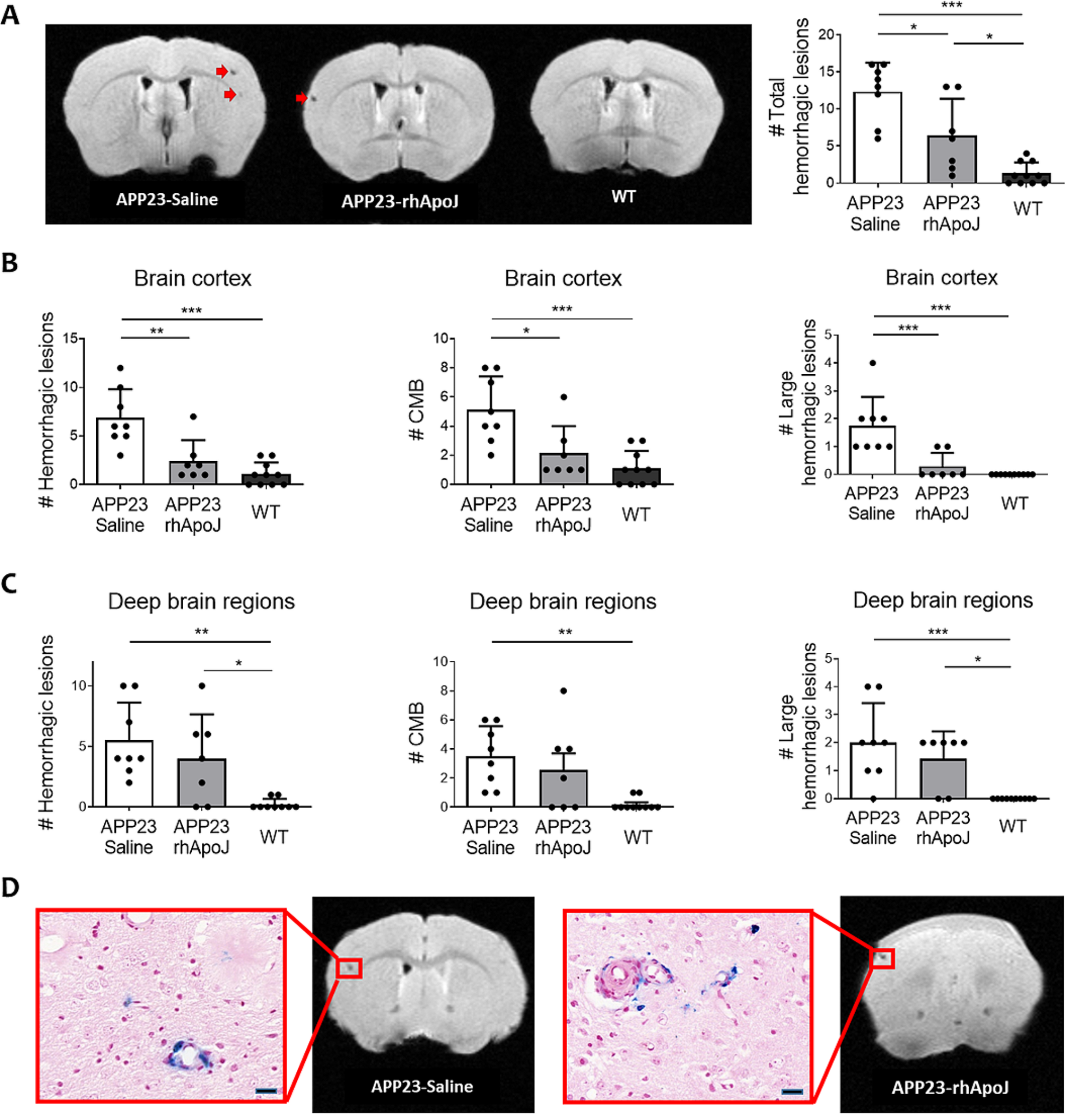
(**A**) Representative T2*-weighted cerebral MRI sections of APP23 mice chronically treated with saline or rhApoJ and WT mice treated with saline. CMB are indicated with red arrows. Graphical representation of the total number of hemorrhagic lesions. (**B**) Graphical representation of the number of hemorrhagic lesions, CMB (50–300 μm diameter) and large hemorrhagic lesions (> 300 μm) in the cortex, and (**C**) in deep brain regions (thalamus and basal ganglia). (**D**) Comparison of cerebral hemorrhagic lesions in T2*-MRI and Prussian blue staining showing iron hemosiderin deposits. The scale bar represents 20 μm. Data are presented as the mean + SD. #: count; *:*p* < 0.05; **:*p* < 0.01; ***:*p* < 0.001

**Fig. 2:**
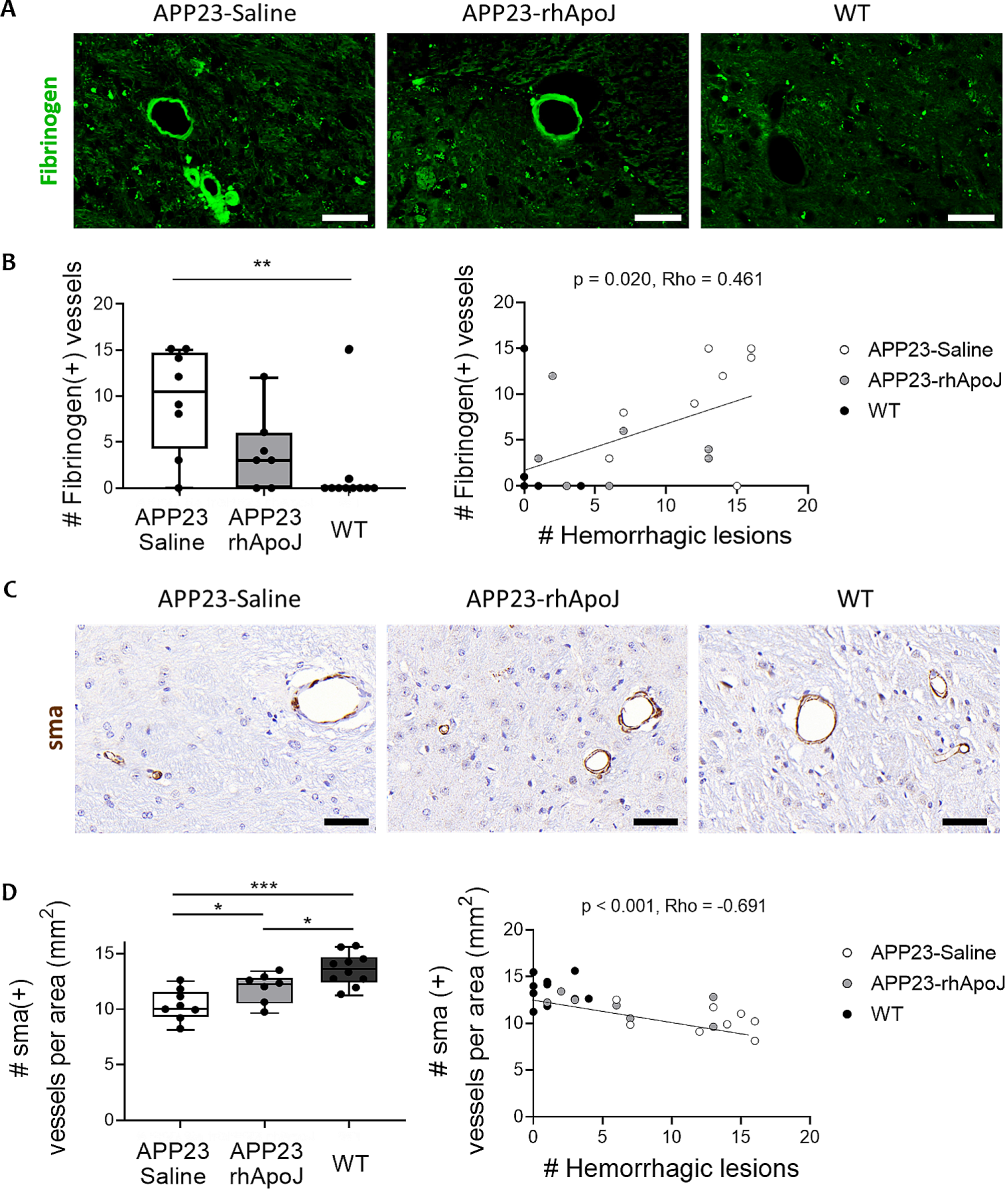
(**A**) Representative images of immunofluorescence staining of fibrinogen in green from brain sections of APP23 mice chronically treated with saline or rhApoJ and WT mice. (**B**) Graphical quantification of fibrinogen-positive cerebral vessels. Correlation between the number of fibrinogen-positive vessels and the number of cerebral hemorrhagic lesions detected by T2*-MRI. (**C**) Representative images of immunohistochemical staining of sma in brown from brain sections of APP23 mice chronically treated with saline or rhApoJ and WT mice. (**D**) Graphical quantification of sma-positive cerebral vessels per mm^2^. Correlation between the number of sma-positive vessels and the number of hemorrhagic lesions. The scale bar represents 50 μm. Data are presented as boxplots. #: count; *:*p* < 0.05; **:*p* < 0.01; ***:*p* < 0.001

Twenty-three-month-old APP23 mice presented a higher number of total cerebral hemorrhagic lesions detected by ex vivo T2*-MRI than WT mice (9.6 ± 5.25 vs. 1.3 ± 1.49, respectively; *p* < 0.001), which is consistent with previous findings [19–22]. More interestingly, APP23 mice chronically treated with rhApoJ showed significantly reduced number of total cerebral hemorrhagic lesions compared to saline-treated APP23 mice (6.43 ± 4.96 vs. 13.38 ± 3.89, respectively; *p* = 0.011) (Fig. 1A). An example of 24 T2*-MRI brain sections from one saline-treated APP23 mouse is provided in Supplemental Fig. 2. Compared with saline treatment, rhApoJ-treated mice presented significantly reduced number of hemorrhages in the cortex (saline-treated: 6.88 ± 2.95 vs. rhApoJ-treated: 2.43 ± 1.15; *p* = 0.002), including CMBs (50–300 μm diameter) (saline-treated: 5.13 ± 2.30 vs. rhApoJ-treated: 2.14 ± 1.86; *p* = 0.012) and larger hemorrhages (> 300 μm) (saline-treated: 1.75 ± 1–04 vs. rhApoJ-treated: 0.29 ± 0.49; *p* = 0.001) (Fig. 1B). However, no differences were observed in the count of hemorrhagic lesions in deep brain areas (Fig. 1C). The mean volume of the hemorrhagic lesions was similar among the groups (saline-treated: 0.041 ± 0.023 mm^3^ vs. rhApoJ-treated: 0.030 ± 0.014 mm^3^; *p* = 0.695) in both the cortex and deep brain areas. Prussian blue staining indicated the presence of blood extravasation in mice, confirming the hemorrhagic lesions detected by T2*-MRI (Fig. 1D). Fibrinogen immunodetection in the brain was evaluated as a marker for blood-brain barrier (BBB) leakage associated with small vessel disease and CAA (Fig. 2A). In this sense, APP23 saline-treated mice presented significantly more fibrinogen-positive vessels than WT mice (10.50 [5.50–14.50] vs. 0.00 [0.00–0.00], respectively; *p* = 0.008), whereas no differences were found between APP23 rhApoJ-treated (3.00 [1.50–5.00]) and WT mouse brains (*p* = 0.457) (Fig. 2B). The number of fibrinogen-positive vessels in the brain significantly correlated (Rho = 0.499, *p* = 0.011) with the number of cerebral hemorrhagic lesions observed by MRI (Fig. 2B). Because CAA is associated with cerebrovascular smooth muscle cell death, which is related to the compromise of the structural integrity and function of blood vessels [3], the number of sma-positive cerebral vessels was also evaluated. APP23 saline-treated mice presented significantly fewer sma-positive vessels (10.30 ± 1.41) than rhApoJ-treated (11.85 ± 1.33, *p* = 0.038) and WT mice (13.52 ± 1.47, *p* < 0.001) (Fig. 2C). Additionally, the number of sma-positive vessels inversely correlated with the number of cerebral hemorrhages detected by MRI (Rho=-0.691, *p* < 0.001) (Fig. 2D).

**Fig. 3:**
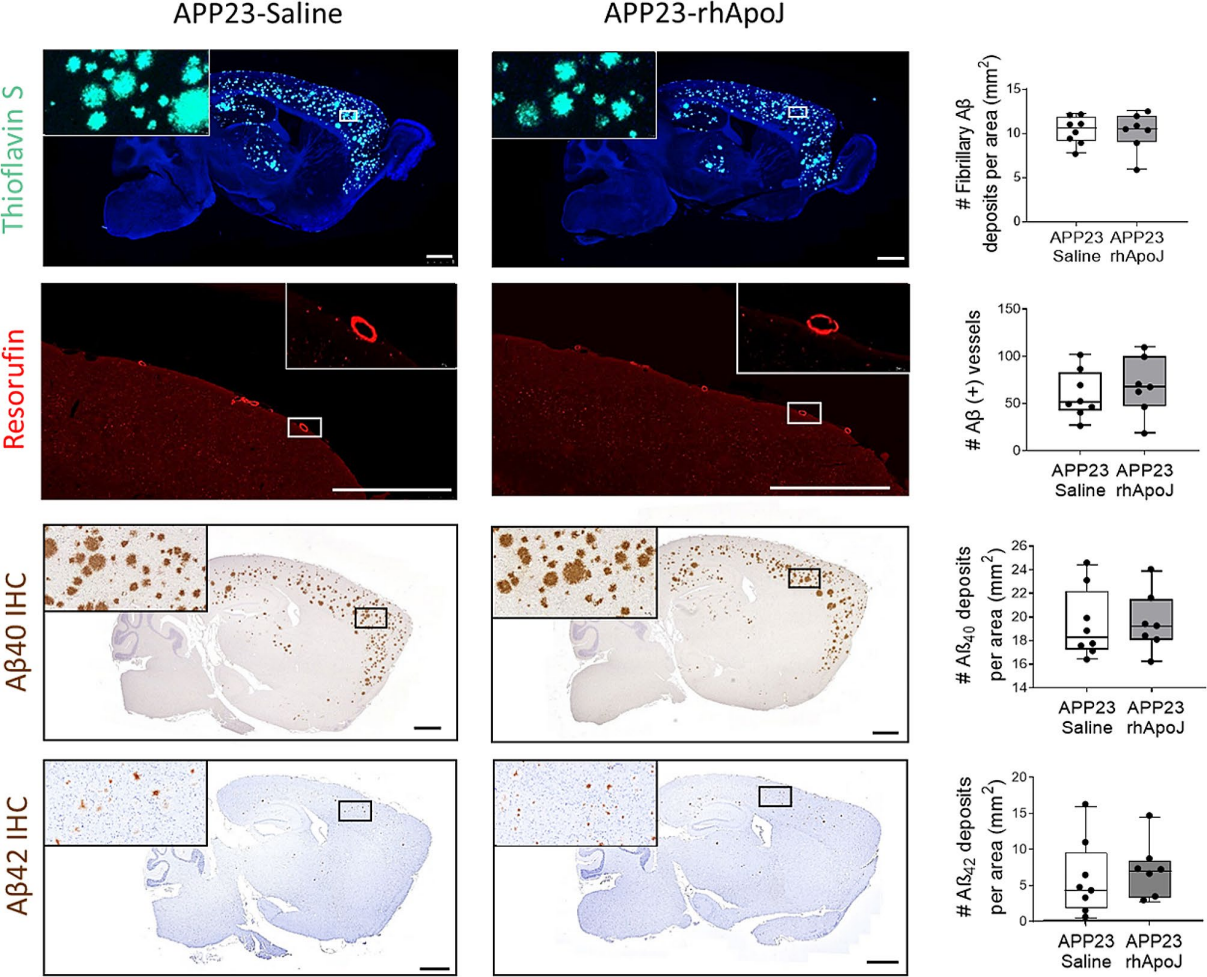
Detection of fibrillar and vascular Aβ in chronically treated APP23 mice. Representative brain sections from APP23 mice chronically treated with saline or rhApoJ and the corresponding graphical quantification in both groups. Vascular Aβ stained with resorufin in red. Fibrillary Aβ stained with thioflavin S in green. Immunohistochemistry staining for Aβ_40_ in brown. Scale bars represent 1000 μm. Data are presented as boxplots. #: count

Aged APP23 transgenic mice are considered a model of CAA for the occurrence of CMBs associated with prominent cerebrovascular Aβ_40_ accumulation [19]. In this regard, vascular Aβ levels were quantified as the number of positive vessels using resorufin staining but also ThS, Aβ_42_ and Aβ_40_ IHC (Supplemental Fig. 3, Supplemental Table 1), but no differences were detected among treatment groups (Fig. 3). Also, no significant differences were observed in the number or size of total brain Aβ deposits and the number of Aβ plaques, whether analyzed by ThS staining or with Aβ40 and Aβ42 IHC (Fig. 3, Supplemental Tables 2 and Supplemental Table 3). Aβ40 levels in CSF of APP23 mice, obtained 3 days after the last administration with rhApoJ or saline, were also similar among treatment groups (Supplemental Table 4). Additionally, considering the known role of ApoJ in lipid metabolism [29], the plasma lipid profile (levels of TChol, LDL-C, HDL-C and triglycerides) of chronically treated mice was analyzed, but no significant differences between groups were found (Supplemental Table 5).

Active microglia are associated with higher pathology severity in human CAA brains [30]. To determine whether the microglial state could be influenced by the rhApoJ treatment and impacted the occurrence of CMB, we analyzed the levels of CD68 (as a marker of phagocytic macrophages and microglia) and Iba1 (as a marker of resident microglia) in brains from mice from group 1. Results showed that cortex from APP23 mice presented higher levels of CD68 than WT brains (Supplemental Fig. 4A), as also occurred for Iba1 (Supplemental Fig. 4B). However, no significant differences were observed between rhApoJ-treated and saline-treated APP23 mouse brains. Lastly, capillary density assessed by Tomato-lectin staining, in brain regions poorly affected by microgliosis, showed no differences among treatment groups (Supplemental Fig. 4C).

**Fig. 4:**
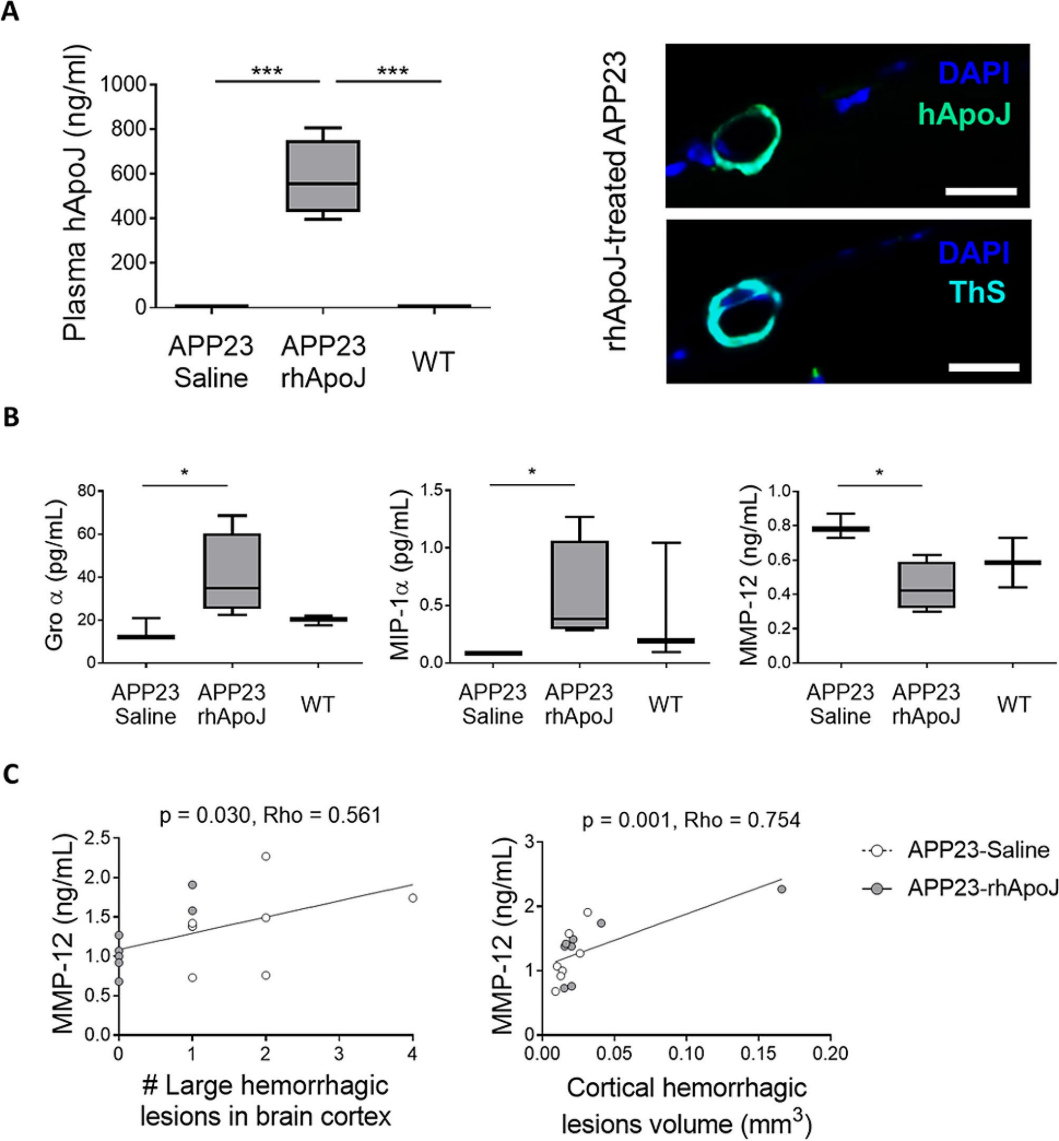
(**A**) Graphical quantification of plasma human ApoJ levels (hApoJ) (ng/mL) and representative image of human ApoJ immunodetection in a brain cortex section from a rhApoJ-treated APP23 mouse. A consecutive brain slice was stained with ThS to confirm the presence of CAA in the vessel. The scale bar represents 20 μm. (**B**) Graphical representation of plasma levels of Groα (pg/mL), MIP-1α (pg/mL), and MMP-12 (ng/mL) in mice from Group 2. Data are presented as boxplots. *:*p* < 0.05; ***:*p* < 0.001. (**C**) Correlation between plasma MMP-12 levels (ng/mL) and the number of large hemorrhagic lesions in the brain cortex (A) and the volume (mm^3^) of cortical hemorrhagic lesions

ApoJ is a short-lived protein with a half-life of 2 h [31]. Because mice from Group 1 were euthanized 3 days after the last dose to avoid potential acute reactions induced by the administration of a recombinant protein, certain molecular mechanisms directly associated with rhApoJ could be masked. To overcome this, a second group of mice (Group 2) was treated and euthanized immediately after administration (30 min time gap) (Supplemental Fig. 1). In contrast to Group 1, in the second group, the presence of rhApoJ was verified in plasma, as well as in some occasional meningeal vessels with CAA in the brain (Fig. 4A). In all cases, rhApoJ was not detected inside the brain, as we previously reported [18]. We next evaluated the peripheral treatment effect regarding potential changes in inflammatory markers and MMPs, which could be related to CMB occurrence. In this sense, rhApoJ-treated mice from Group 2 presented significantly higher plasma levels of the chemokines Groα (saline-treated: 12.14 [11.78–16.56] pg/mL vs. rhApoJ-treated: 34.73 [28.33–51.82] pg/mL, *p* = 0.035) and macrophage inflammatory protein (MIP)-1α (saline-treated: 0.08 [0.08–0.08] pg/mL vs. rhApoJ-treated: 0.38 [0.30–0.86] pg/mL, *p* = 0.035) than saline-treated APP23 mice (Fig. 4B, Supplemental Table 6). Regarding plasma MMPs levels, they remained similar between groups except for MMP-12. Specifically, rhApoJ-treated mice showed significantly lower levels of circulating MMP-12 than saline-treated mice (0.44 ± 0.14 ng/mL vs. 0.79 ± 0.07 ng/mL, respectively; *p* = 0.046) (Fig. 4B, Supplemental Table 6). To further investigate these findings, plasma levels from Group 1 were also analyzed. While no differences between treatment groups were detected, significant correlations between plasma MMP-12 levels and the number of large hemorrhagic cortical lesions (Rho = 0.561, *p* = 0.030) (Fig. 4C) and the volume of cortical hemorrhagic lesions (Rho = 0.754, *p* = 0.001) (Fig. 4C) were found in APP23 mice, as determined by ex vivo MRI.

**Fig. 5:**
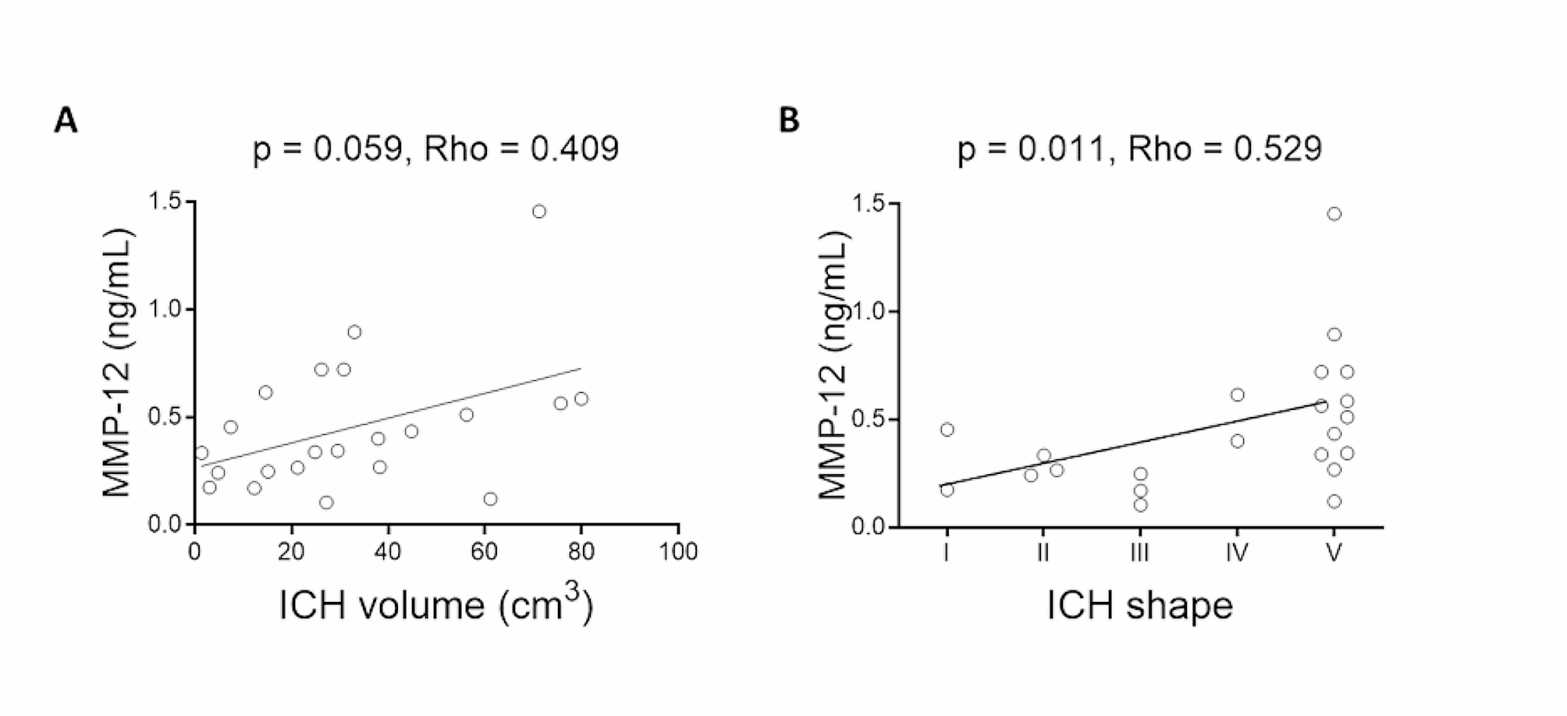
(**A**) Correlation between MMP-12 plasma levels and ICH volume (cm^3^) and (**B**) the rate of ICH shape from a human cohort presenting with acute lobar ICH.

To expand the results obtained in mice, and given the previously described involvement of MMP-12 in BBB leakage and neuroinflammation in different neurological experimental models [32–34], we further explored the possible association between circulating MMP-12 levels and cerebral hemorrhagic load in humans. For this purpose, we evaluated MMP-12 plasma levels in lobar ICH patients, commonly associated with an underlying CAA pathology. A descriptive summary of the studied population is provided in Supplemental Table 7. Interestingly, in this cohort, circulating MMP-12 levels were correlated with ICH volume (Fig. 5A), ICH shape (Fig. 5B) and prothrombin time in the acute phase (Supplemental Table 8). Associations between plasma MMP-12 level and ICH shape and volume remained significant after adjusting for sex, age, and prothrombin time by linear regression (B = 0.006 [0.000–0.011], *p* = 0.040 for ICH volume; and B = 0.095 [0.007–0.183], *p* = 0.036 for ICH shape) (Supplemental Table 9).

## Discussion

Apolipoproteins, specifically Apolipoprotein E (ApoE) and ApoJ, are closely related to AD and CAA. Both ApoE and ApoJ co-deposit with cerebral Aβ in the parenchyma and vasculature [13] and are multifunctional proteins involved in Aβ aggregation and clearance and other processes, such as lipid metabolism and neuroinflammation [29, 35]. APOE is the most important genetic risk factor for sporadic AD and CAA [5, 36–38], while CLU, which encodes ApoJ, has also been extensively linked to AD [39–41]. Furthermore, we observed that CLU single nucleotide polymorphisms (SNPs) were associated with the presence of lobar microbleeds in CAA cohorts [42, 43]. In this context, we previously studied the role of circulating ApoJ in AD and CAA at a preclinical level. Specifically, we tested the effect of the subchronic administration of rhApoJ in fifteen-month-old APP23 mice, observing a reduction in Aβ parenchymal and vascular levels, along with a decrease in the neurodegeneration associated with the model [18]. However, the impact of the treatment on cerebrovascular health and the risk of microbleeds occurrence was not evaluated because such vascular lesions appear at an advanced stage of the pathology [20, 22]. Therefore, in the present study, older APP23 mice were chronically treated with the same dose of rhApoJ or saline, and the presence of cerebral hemorrhagic lesions was determined through ex vivo T2*-weighted MRI.

The main finding of our study was that increasing the levels of peripheral ApoJ through chronic treatment with rhApoJ had an impact on relevant clinical endpoints associated with CAA, such as the cerebral hemorrhagic load. Specifically, rhApoJ-treated APP23 mice presented a significant reduction in the number of cerebral hemorrhagic lesions detected by MRI, which were associated with BBB leakage detected as extravasated fibrinogen in brain vessels. In CAA, BBB molecular changes have been well demonstrated [44–46], suggesting that BBB damage can result in CMB development [47–49]. Additionally, it is known that APP23 mice experience a loss of sma in brain vessels [50]. In this sense, rhApoJ treatment prevented the loss of sma-positive brain vessels in APP23 mice, suggesting an improvement in the cerebrovascular functional status. Because the reduction in the number of hemorrhagic lesions was not observed when the analysis was carried out in deep brain areas, our results suggest a specific effect of the treatment on those superficial arteries more commonly affected by CAA. However, the observed reduction in cerebral hemorrhagic load in aged APP23 mice due to rhApoJ treatment could not be attributed to the modulation of vascular and/or parenchymal fibrillar Aβ levels, since these parameters remained unaltered by the treatment. However, we cannot completely rule out that levels of soluble Aβ forms can be influenced by rhApoJ in aged APP23 mice, despite no differences in Aβ40 levels in the CSF were detected. Regarding Aβ levels and CAA load, in our previous study, we observed that the subchronic rhApoJ-treatment of middle-aged APP23 mice reduced soluble and insoluble Aβ levels, as well as parenchymal Aβ-plaque size and the number of Aβ-positive cerebral vessels [18]. In fact, several studies have observed that the genetic alteration of CLU or the administration of ApoJ-mimetic peptides in AD transgenic mouse models are able to modulate the Aβ load and/or its intracerebral distribution [51–55]. Altogether, these studies highlight a relevant function of ApoJ in regulating Aβ aggregation and clearance from the brain. A possible reason for the presumable discordance with our present study would be based on the age of the experimental mice used. In the current study, the Aβ-associated pathology was already well established and saturated with few possibilities to modulate the levels of fibrillary Aβ. Therefore, because rhApoJ treatment did not show an impact in terms of Aβ modulation in aged mice, it can be speculated that the beneficial effects of rhApoJ may be due to mechanisms related to an improvement of the cerebrovascular condition, that would be translated into a direct effect on Aβ mobilization through perivascular drainage pathways in younger mice, but a lower occurrence of CMB in older mice.

Interestingly, after different time points of administration (3 days or 30 min prior to euthanasia), rhApoJ was not detected inside the brain, which is consistent with our previous study [18], and suggest that its mechanism of action on brain vasculature operates through the periphery. Indeed, when mouse tissue was obtained immediately after administration, rhApoJ was found in plasma and co-deposing with fibrillar Aβ in occasional meningeal vessels, suggesting a transitory presence of rhApoJ on affected vessels. Since the human recombinant protein was only detected in leptomeningeal vessels, we speculate that the effect of the protein could be preferentially directed to more superficial brain vessels, which would explain the effect observed regarding the reduction of cortical CMBs rather than deep CMBs by rhApoJ chronic treatment. Indeed, the precise mechanism by which peripheral rhApoJ reduced the number of CMB in APP23 mice in the present study was not completely elucidated. However, the analysis of different circulating inflammatory markers shed light on this question. Notably, we observed a significant increase in plasma Groα and MIP-1α levels in rhApoJ-treated APP23 mice when compared to saline-treated APP23 mice, molecules previously proposed to enhance monocyte migration to the brain in an AD context [56, 57]. Monocytes play an important role after cerebral hemorrhage, as they can potentiate inflammation but also contribute to hematoma removal and tissue healing [58]. On the other hand, we found that MMP-12 plasma levels were significantly reduced in rhApoJ-treated APP23 mice. Furthermore, MMP-12 plasma levels were significantly associated with the number of large cortical hemorrhages and the volume of cortical hemorrhagic lesions in APP23 mice. Several MMPs and tissue inhibitors of metalloproteinases (TIMPs)  have been previously associated with CAA and are proposed to contribute to vascular damage leading to brain bleeding [59, 60]. Interestingly, increased expression of MMP family members, associated with the activation of perivascular macrophages around vascular Aβ deposits, have been recently implicated in microhemorrhages induced by Aβ immunotherapy in mice [61]. In particular, we hypothesize that the effect of rhApoJ on the cerebrovasculature may be associated with its capacity to partially modulate circulating MMP-12 levels, since an established link between MMP-12 and BBB damage in experimental models has been previously proposed [32, 34]. Indeed, MMP-12 induces BBB damage after cerebral ischemia and its levels are elevated after ICH in rodent models, which has a negative impact on sensorimotor function [32–34]. In addition, MMP-12 is implicated in the pathogenesis of many inflammatory diseases, and its secretion by microglia is significantly upregulated by Aβ_42_ treatment in vitro [62]. Actually, MMP-12 has been proposed as a promising therapeutic target for neurological diseases, including ICH [63]. In our study, we observed, for the first time, a significant association between plasma MMP-12 levels and the volume of lobar ICH, commonly associated to a CAA etiology, in a human cohort. Also, we found a significant association between MMP-12 plasma levels and a more irregular shape of ICH, which is a neuroimaging parameter associated with hemorrhage expansion [64]. Thus, we suggest that plasma MMP-12 levels could have an impact on BBB alteration and the brain hemorrhagic load in a cerebral β-amyloidosis context. The mechanisms by which ApoJ can influence MMP-12 levels remain elusive. However, prior research has shown that ApoJ can reduce the levels and activity of other MMP family proteins, such as MMP-9, MMP-3 and MMP-7 [65–67]. In addition, it is well known that ApoJ is an inhibitor or the complement [68, 69], which is activated by vascular Aβ pathology [70]. In this sense, it has been reported that different components of the complement cascade upregulate the production and release of several MMPs, including MMP-12 [71, 72]. Therefore, it could be speculated a downregulation of MMP-12 through the inactivation of the complement pathway induced by high doses of ApoJ.

In summary, our study shows that the chronic treatment with rhApoJ reduces the number of cerebral microhemorrhages in an aged β-amyloidosis mouse model independently of a direct modulation of fibrillar brain Aβ levels. These results might be important in the context of the new passive immunotherapy era in AD and open the possibility to combine the use of Aβ monoclonal antibodies with therapies that promote an increase of circulating ApoJ, with the aim to protect the cerebrovasculature and reduce the risk of ARIA. Furthermore, we consider that our data contribute to expanding the possible implication of ApoJ and MMP-12 in BBB leakage associated to CAA with future implications for the diagnosis and treatment of CAA.

Our study presents some limitations, primarily based on the fact of working with aged mice, which are associated with higher mortality [73] and consequently resulted in a relatively small sample size. All mice used were females, as we previously observed that female APP23 mice exhibited higher brain Aβ accumulation [19], assuming a more severe Aβ pathology than male mice. It is important to acknowledge this limitation, and future investigations should encompass the inclusion of aged male APP23 mice to comprehensively address this aspect. Another limitation is the lack of functional analysis after the rhApoJ chronic treatment in APP23 mice. In this regard, a previous study reported that the treatment with a synthetic peptide of ApoJ improved the cognitive function in 5xFAD mice, an accelerated model of cerebral β-amyloidosis [51]. Thus, further studies testing the safety and efficacy of rhApoJ treatment using different models of AD will be relevant to determine its impact on behavior and cognition. Additionally, a basal MRI to assess the number of CMBs before treatment was not performed, mainly because of the risks associated with long periods of anesthesia in aged mice. Another limitation of our study would be that hemorrhagic lesions have been only quantified by T2*-MRI. However, Prussian blue staining was used to demonstrate that the hypointensities observed in T2*-MRI corresponded to cerebral blood extravasation. Furthermore, a strong correlation between the number of CMBs identified by classical histological techniques and T2*-MRI has been previously demonstrated [74]. Finally, WT mice were not treated with rhApoJ since the principal aim of the study was to determine whether the number of CMB associated with CAA was impacted by the treatment with the recombinant chaperone respect to the saline treatment. However, future experiments should contemplate to include this group of rhApoJ-treated WT mice to further understand the age-related molecular pathways associated with treatment. Regarding the human population, our information is restricted to CT scans since MRIs were not available for all patients. Finally, because of the exploratory nature of this paper, a replication study was not performed and *p* values < 0.05 were considered statistically significant.

## Conclusions

Our results show that circulating rhApoJ presence reduces the occurrence of cerebral microhemorrhages in a β-amyloidosis mouse model. We propose that peripheral rhApoJ treatment could partially prevent the BBB fragility and brain hemorrhagic load associated with CAA by modulating circulating MMP-12 levels. We suggest that treatments based on the upregulation of ApoJ offer a noninvasive therapeutic opportunity to ameliorate the cerebrovascular pathology associated with Aβ brain accumulation.

### Electronic supplementary material

Below is the link to the electronic supplementary material.

Supplementary Material 1

## Data Availability

The datasets used and/or analysed during the current study are available from the corresponding author on reasonable request.

## Abbreviations

Aβ: Amyloidβ
AD: Alzheimer’s disease
ApoJ: Apolipoprotein J
APP: Amyloid precursor protein
B: Slope
BBB: Blood-brain barrier
BCA: Bicinchoninic acid assay
BSA: Bovine Serum Albumin
CAA: Cerebral amyloid Angiopathy
CD68: Cluster of Differentiation 68
CI: Confidence interval
CMB: Cerebral microbleeds
CSF: Cerebrospinal fluid
CT: Computed tomography
DAB: Diaminobenzidine
ELISA: Enzyme-linked immunosorbent assay
EDTA: Ethylenediaminetetraacetic acid
FBS: Fetal Bovine Serum
FLPC: fast protein liquid chromatography
HEK293T: Human embryonic kidney 293 T cells
HDL-C: High-density lipoprotein-cholesterol
HUVH: Hospital Universitari Vall d’Hebron
Iba1: Ionized calcium-binding adapter molecule 1
IHC: Immunohistochemistry
INR: International Normalized Ratio
ICH: Intracerebral hemorrhage
LDL-C: Low-density lipoprotein-cholesterol
MIP: Macrophage inflammatory protein
MRI: Magnetic resonance imaging
MMP: Matrix metalloproteinase
ON: Overnight
PFA: Paraformaldehyde
PBST: PBS 0.2% Triton
rhApoJ: Recombinant human ApoJ
RT: Room temperature
sma: Smooth muscle actin
SNPs: Single nucleotide polymorphisms
TChol: Total cholesterol
TGs: Triglycerides
ThS: Thioflavin S
UAB: Universitat Autònoma de Barcelona
WT: Wild-type
