# Supplementary material for: The presence of circulating human apolipoprotein J reduces the occurrence of cerebral microbleeds in a transgenic mouse model with cerebral amyloid angiopathy
Source: Alzheimers Res Ther. 2024 Jul 29;16:169. doi: 10.1186/s13195-024-01541-5 (PMC11285315; doi:10.1186/s13195-024-01541-5)
Supplement: Supplementary file 1 — Supplementary Material 1 [file 13195_2024_1541_MOESM1_ESM.docx]

**Supplementary Materials**

**Supplemental Figure 1**


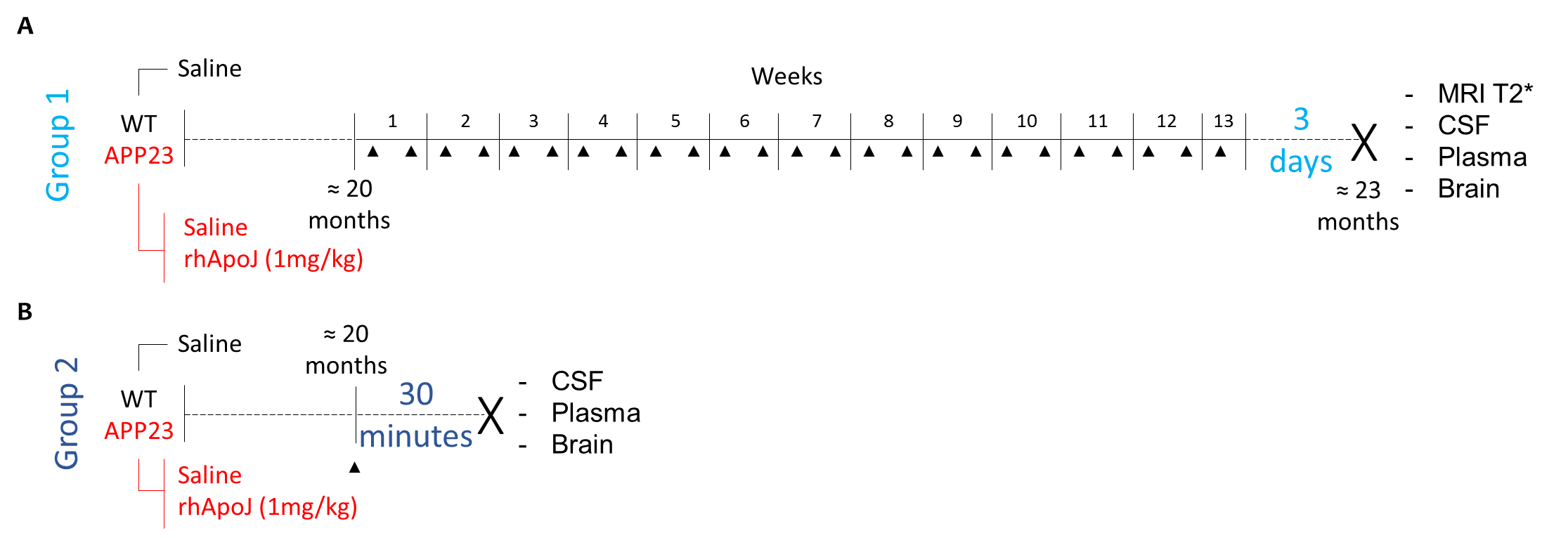


**Supplemental Figure 1.** Study timeline for A) Group 1 and B) Broup 2. Black triangles represent each administration. Crosses in the diagrams represent euthanasia.

**Supplemental Figure 2**


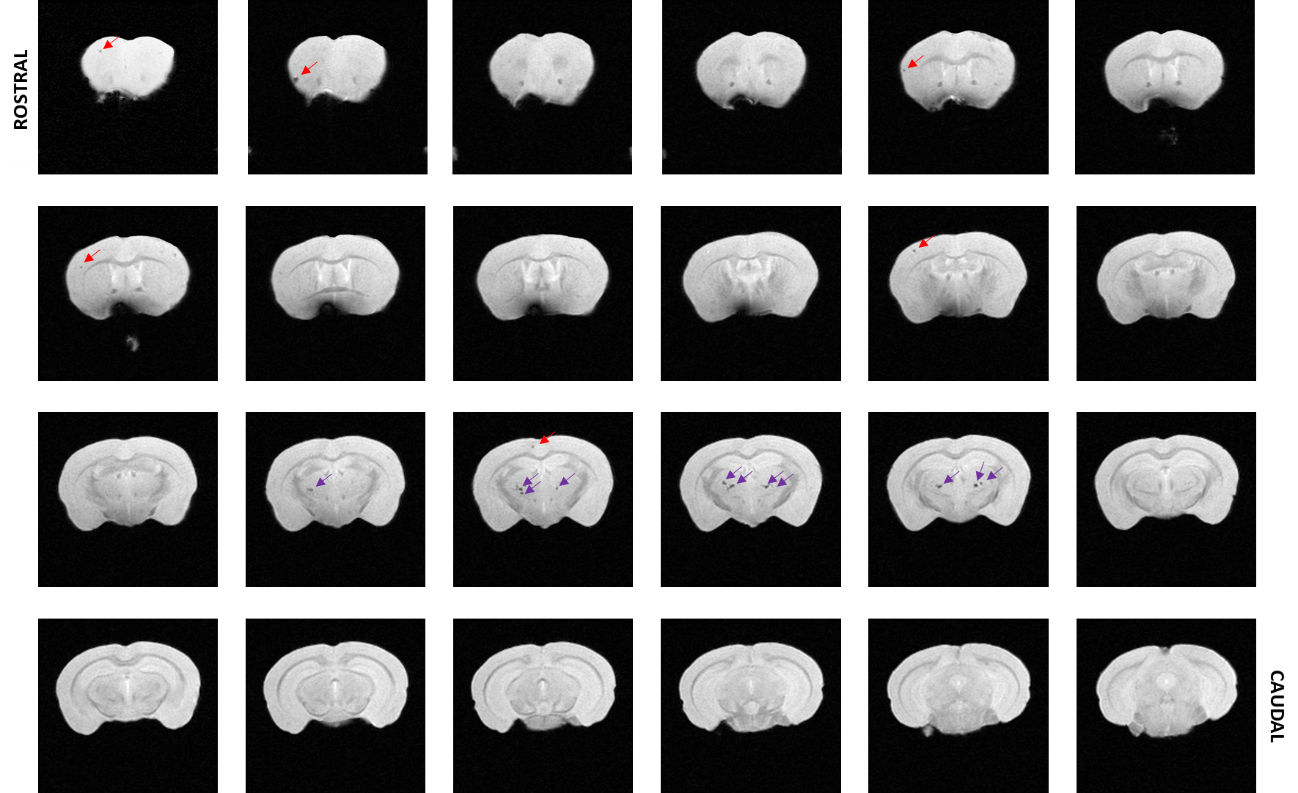


**Supplemental Figure 2.** Representation of T2*-weighted MRI cerebral sections of 23 months old chronically saline-treated APP23 mice from rostral to caudal. Cortical CMB are indicated with red arrows and deep CMB with purple arrows.

**Supplemental Figure 3**

**
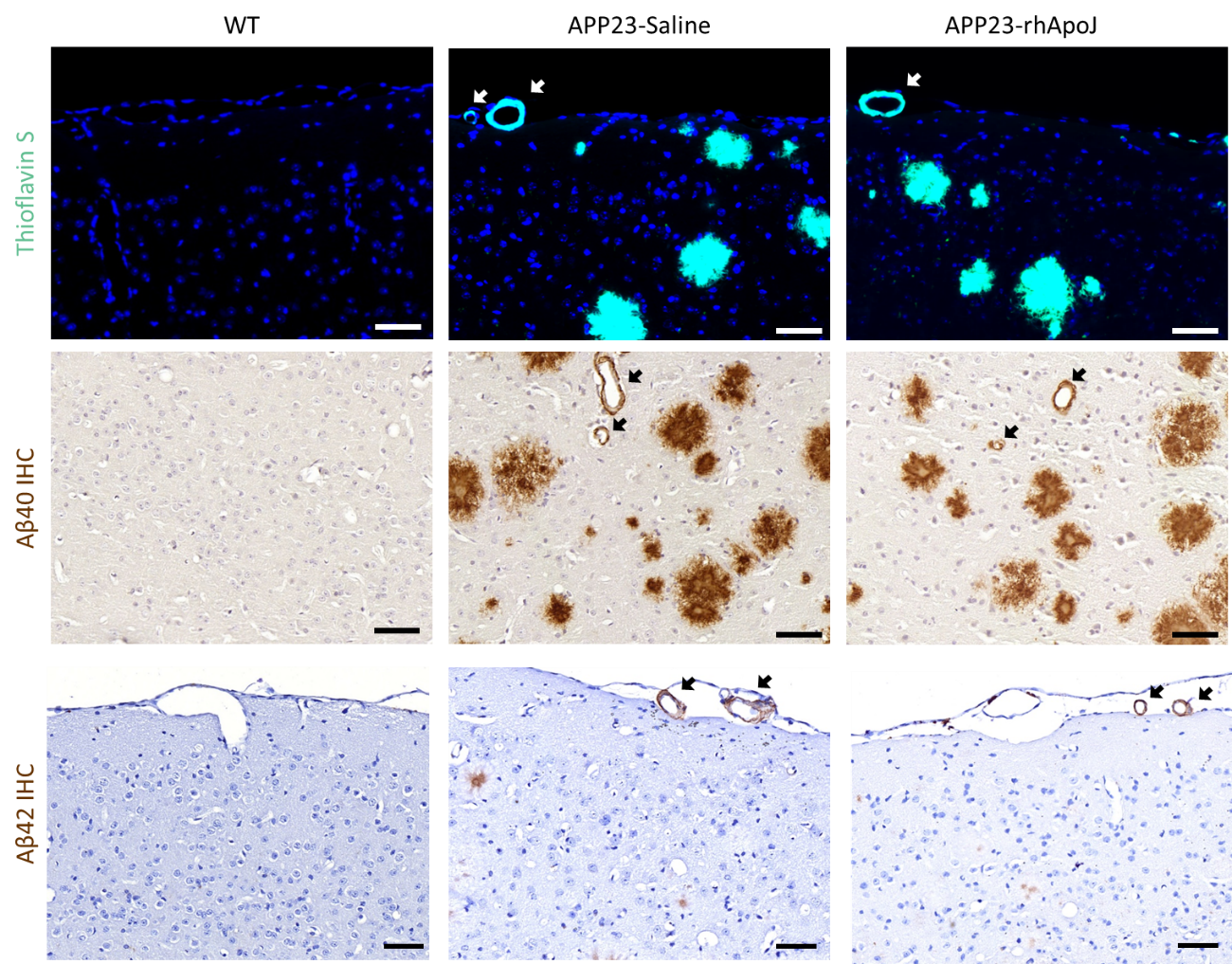
**

**Supplemental Figure 3.** Representation of vascular Aβ staining (arrows) in chronically treated APP23 mice by Thioflavin S in green and immunohistochemistry staining for Aβ40 and Aβ42 in brown. Scale bars represent 50 µm.

**Supplemental Figure 4**

**
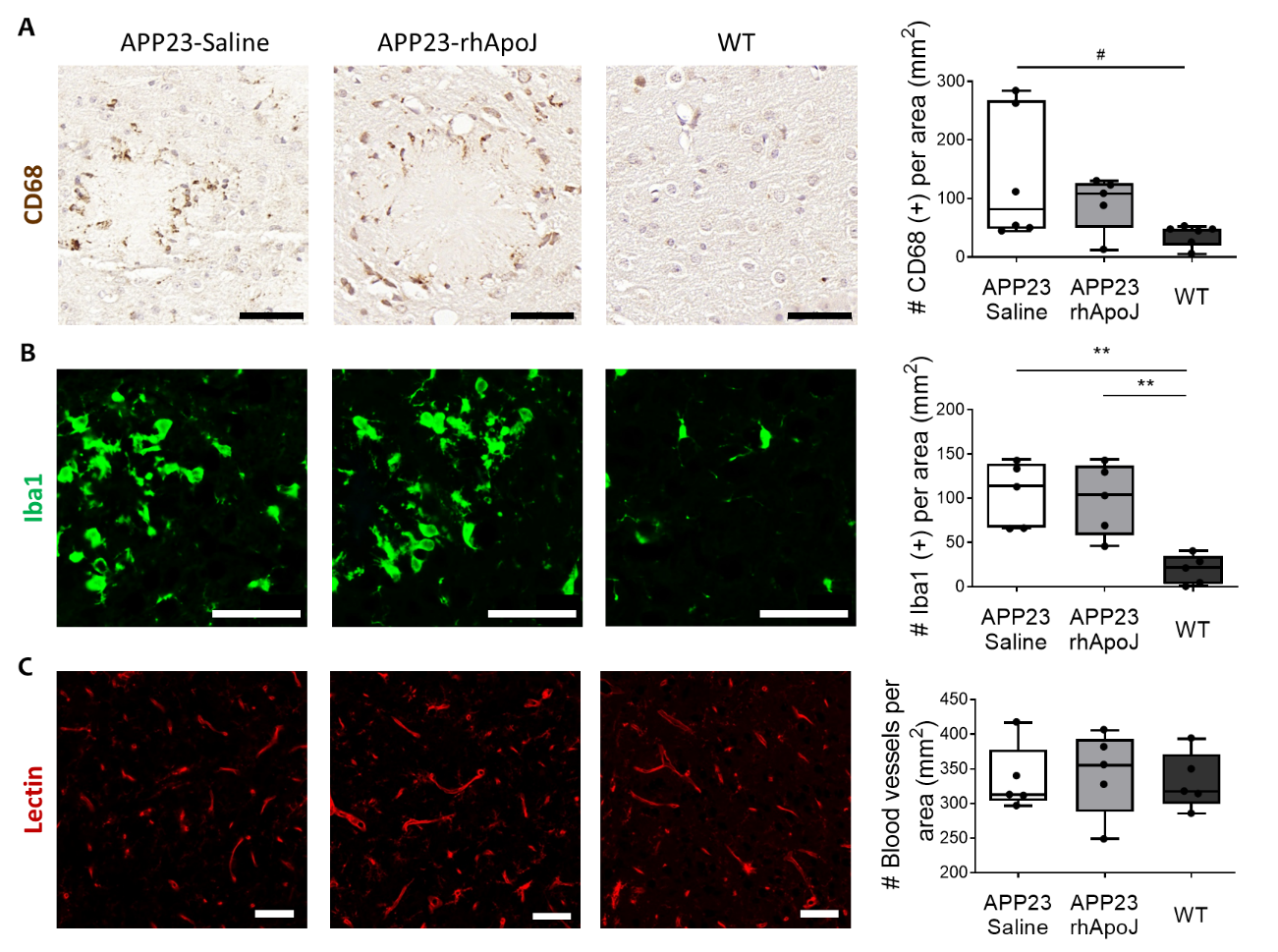
**

**Supplemental Figure 4.** Representative images of brains from APP23 mice, chronically treated with saline or rhApoJ, and WT mice. **A)** Immunohistochemistry against mouse CD68, indicative of phagocytic activity, is shown in brown. Graphical quantification of positive CD68 signal in the cortex. **B)** Immunofluorescence against Iba1, indicative of resident microglia, is shown green. Graphical quantification of positive Iba1 signal in the cortex. **C)** Tomato-lectin staining is shown in red. Graphical quantification of capillary density (# blood vessels per area) in the striatum. The bar scale represents 50 µm. Data are presented as boxplots. #:p<0.1 **:p<0.01.

**Supplemental Table 1.** Association of the number of Aβ-positive brain vessels in APP23 mice (Group 1), which were chronically treated with rhApoJ or saline.

|  | **Location** | **APP23-Saline**  n=8 | **APP23-rhApoJ**  n=7 | **p** |
| --- | --- | --- | --- | --- |
| **Resorufin-positive vessels** | Meninges | 14.63 ± 8.07 | 12.14 ± 10.04 | 0.604 |
|  | Cortex | 7.50 [3.00–17.50] | 12.00 [2.00–29.00] | 0.779 |
|  | Meninges and cortex | 23.88 ± 12.98 | 26.00 ± 22.49 | 0.823 |
|  | Hippocampus | 1.00 [0–4.50] | 0 [0–3.00] | 0.463 |
|  | Thalamus | 23.38 ± 8.45 | 32.71 ± 11.13 | 0.088 |
|  | Total | 59.63 ± 24.96 | 68.29 ± 30.72 | 0.557 |
| **ThS-positive  vessels** | Meninges | 23.50 ± 5.53 | 20.86 ± 1.95 | 0.238 |
|  | Cortex | 7.50 [3.75–13.25] | 13.00 [1.00–20.00] | 0.867 |
|  | Meninges and cortex | 33.88 ± 13.32 | 34.00 ± 12.33 | 0.985 |
|  | Hippocampus | 2.00 [1.25–3.00] | 1.00 [0–5.00] | 0.336 |
|  | Thalamus | 37.5 ± 28.13 | 42.71 ±17.26 | 0.678 |
|  | Total | 73.00  [56.50–94.25] | 98.00  [87.00–100.00] | 0.336 |
| **Aβ_40_-positive  vessels** | Meninges | 33.88 ± 13.58 | 29.71 ± 10.40 | 0.522 |
|  | Cortex | 31.38 ± 26.34 | 33.14 ± 22.67 | 0.892 |
|  | Meninges and cortex | 65.25 ± 33.04 | 62.86 ± 30.93 | 0.888 |
|  | Hippocampus | 2.00  [0.00–10.00] | 2.00  [1.00–6.50] | 0.867 |
|  | Thalamus | 27 .00  [17.00–42.50] | 22.00  [17.00–52.00] | 0.779 |
|  | Total | 116.00  [82.50–133.50] | 125.00  [95.50–147.00] | 0.779 |
| **Aβ_42_-positive  vessels** | Meninges | 37.75 ± 10.71 | 31.00 ± 8.98 | 0.213 |
|  | Cortex | 16.38 ± 11.48 | 25.00 ± 16.84 | 0.262 |
|  | Meninges and cortex | 55.88 ± 18.95 | 56.00 ± 22.97 | 0.991 |
|  | Hippocampus | 1.00 [1.00–2.00] | 2.00 [1.00–2.00] | 0.757 |
|  | Thalamus | 8.00 [7.00–10.50] | 12.00 [7.00–15.50] | 0.483 |
|  | Total | 77.88 ± 26.24 | 80.86 ± 32.46 | 0.847 |

**Supplemental Table 2.** Aβ parenchymal levels analyzed by Ths and Aβ_40_ IHC in APP23 mice (Group 1), which were chronically treated with rhApoJ or saline.

|  |  | **Brain location** | **APP23-Saline**  n=8 | **APP23-rhApoJ**  n= 7 | **p** |
| --- | --- | --- | --- | --- | --- |
| **ThS-positive**  **deposits** | **# Fibrillary Aβ**  **(deposits/mm^2^)** | Cortex | 21.86 ± 3.17 | 20.48 ± 4.80 | 0.517 |
|  |  | Hippocampus | 15.04 ± 3.54 | 16.72 ± 4.15 | 0.414 |
|  |  | Thalamus | 6.99 ± 2.29 | 6.91 ± 3.27 | 0.957 |
|  |  | Total | 10.38 ± 1.57 | 10.21 ± 2.20 | 0.867 |
|  | **Percentage of fibrillary Aβ (%)** | Cortex | 9.73 ±2.42 | 8.75 ± 2.45 | 0.449 |
|  |  | Hippocampus | 6.39 ± 2.38 | 6.57 ± 2.3 | 0.879 |
|  |  | Thalamus | 3.49 ± 1.43 | 2.89 ± 1.60 | 0.459 |
|  |  | Total | 4.63 ± 1.11 | 4.31 ± 1.3 | 0.608 |
|  | **Average Size of fibrillary Aβ deposits**  **(µm^2^)** | Cortex | 4459.66 ± 902.33 | 4301.40 ± 1101.7 | 0.764 |
|  |  | Hippocampus | 4288.15 ± 1441.66 | 3965.34 ± 1166.91 | 0.645 |
|  |  | Thalamus | 5067.77 ± 1906.77 | 4154.20 ± 1228.76 | 0.298 |
|  |  | Total | 4465.10 ± 824.22 | 4186.05 ± 1001.73 | 0.564 |
| **Aβ_40_–positive deposits** | **# Aβ_40_**  **(deposits/mm^2^)** | Cortex | 35.91 ± 5.17 | 36.10 ± 3.19 | 0.933 |
|  |  | Hippocampus | 22.12 ± 5.57 | 25.15 ± 5.98 | 0.328 |
|  |  | Thalamus | 17.00 ± 11.58 | 17.16 ± 7.20 | 0.978 |
|  |  | Total | 19.19 ± 2.77 | 19.12 ± 2.02 | 0.957 |
|  | **Percentage of Aβ_40_ (%)** | Cortex | 7.86 ± 1.73 | 8.41 ± 2.00 | 0.577 |
|  |  | Hippocampus | 4.91 ± 2.50 | 6.00 ±1.45 | 0.333 |
|  |  | Thalamus | 1.56 [0.98–1.90] | 2.23 [1.31–4.23] | 0.336 |
|  |  | Total | 3.84 ± 0.72 | 4.14 ± 1.19 | 0.557 |
|  | **Average Size of Aβ_40_ deposit (µm^2^)** | Cortex | 2180.46 ± 308.50 | 2328.88 ± 522.79 | 0.508 |
|  |  | Hippocampus | 2194.21 ± 849.41 | 2407.52 ± 339.38 | 0.546 |
|  |  | Thalamus | 1024.57 ± 528.90 | 1510.57 ± 928.77 | 0.227 |
|  |  | Total | 2006.87 ± 279.29 | 2145.32 ± 500.46 | 0.512 |

| **Aβ_42_–positive deposits** | **# Aβ_42_**  **(deposits/mm^2^)** | Cortex | 9.41 ± 5.69 | 9.28 ± 3.74 | 0.959 |
| --- | --- | --- | --- | --- | --- |
|  |  | Hippocampus | 6.87 ± 4.86 | 8.14 ± 4.51 | 0.610 |
|  |  | Thalamus | 2.53 ± 1.24 | 3.31 ± 1.98 | 0.370 |
|  |  | Total | 5.86 ± 3.17 | 5.59 ± 1.51 | 0.834 |
|  | **Percentage of Aβ_42_ (%)** | Cortex | 0.16 ± 0.12 | 0.19 ± 0.11 | 0.615 |
|  |  | Hippocampus | 0.15 ± 0.13 | 0.16 ± 0.10 | 0.913 |
|  |  | Thalamus | 0.05 ± 0.02 | 0.07 ± 0.05 | 0.257 |
|  |  | Total | 0.10 ± 0.07 | 0.11 ± 0.04 | 0.811 |
|  | **Average Size of Aβ_42_ deposit (µm^2^)** | Cortex | 158.42 ± 55.77 | 199.83 ± 52.06 | 0.163 |
|  |  | Hippocampus | 203.78 ± 110.41 | 198.63 ± 55.97 | 0.913 |
|  |  | Thalamus | 211.97 ± 114.96 | 202.22 ± 131.69 | 0.881 |
|  |  | Total | 164.98 ± 43.18 | 198.63 ± 48.92 | 0.180 |

**Supplemental Table 3**. Association of the number of Aβ-plaques in APP23 mice (Group 1), which were chronically treated with rhApoJ or saline.

|  |  | **Brain location** | **APP23-Saline**  n=8 | **APP23-rhApoJ**  n=7 | **p** |
| --- | --- | --- | --- | --- | --- |
| **Aβ Plaques** | **# ThS-positive Aβ plaques**  **(plaques/mm^2^)** | Cortex | 19.29  [16.82–22.10] | 18.83  [15.06–22.12] | 0.867 |
|  |  | Hippocampus | 14.00 ± 3.90 | 15.83 ± 4.17 | 0.396 |
|  |  | Thalamus | 0.72  [0.00–2.63] | 0.00  [0.00–0.71] | 0.536 |
|  |  | Total | 7.81 ± 2.01 | 7.25 ± 2.66 | 0.652 |
|  | **# Aβ_40_ plaques**  **(plaques/mm^2^)** | Cortex | 30.88 ± 3.89 | 31.40 ± 4.52 | 0.816 |
|  |  | Hippocampus | 21.91 ± 5.35 | 25.39 ± 6.49 | 0.311 |
|  |  | Thalamus | 4.35 ± 5.91 | 5.91 ± 3.49 | 0.554 |
|  |  | Total | 15.26 ± 1.82 | 15.30 ± 2.11 | 0.970 |
|  | **# Aβ_42_ plaques**  **(plaques/mm^2^)** | Cortex | 7.94 ± 5.39 | 6.73 ± 4.05 | 0.635 |
|  |  | Hippocampus | 6.42 ± 4.84 | 7.73 ± 4.73 | 0.607 |
|  |  | Thalamus | 0.00  [0.00–1.03] | 0.00  [0.00–0.76] | 1.00 |
|  |  | Total | 3.76  [2.42–7.25] | 4.83  [2.16–5.00] | 0.817 |

**Supplemental Table 4**. Aβ_40_ CSF levels in APP23 mice (Group 1), which were chronically treated with rhApoJ or saline.

|  | **APP23-Saline**  n=7 | **APP23-rhApoJ**  n= 6 | **p** |
| --- | --- | --- | --- |
| Aβ_40_ CSF (pg/mL) | 21152.33 ± 11210.49 | 24932.70 ± 3909.55 | 0.457 |

**Supplemental Table 5**. Association of fasting lipid metabolism in APP23 and WT mice (Group 1).

|  | **WT**  n=10 | **APP23-Saline**  n=8 | **APP23-rhApoJ**  n= 7 | **p** |
| --- | --- | --- | --- | --- |
| **Total Cholesterol (mg/dL)** | 56.00 ± 16.69 | 57.20 ± 12.91 | 50.00 ± 15.90 | 0.765 |
| **LDL-C (mg/dL)** | 92.10 ± 10.97 | 80.00 ± 12.39 | 81.00 ± 9.83 | 0.105 |
| **HDL-C (mg/dL)** | 28.60 ± 9.50 | 25.40 ± 10.69 | 25.25 ± 12.45 | 0.794 |
| **Triglycerides (mg/dL)** | 66.37 ± 24.89 | 66.68 ± 25.50 | 59.08 ± 30.36 | 0.882 |

**Supplemental Table 6.** Association of plasma levels of inflammation markers and MMPs with treatment in APP23 mice and WT mice (Group 2).

|  | **APP23-Saline**  n=3 | **APP23-rhApoJ**  n=4 | **WT**  n=3 | **p** |
| --- | --- | --- | --- | --- |
| Groα (pg/mL) | 12.14  [11.78–16.56] | 34.73 *****  [28.33–51.82] | 20.21  [18.88–21.04] | **0.030** |
| IL-1β (pg/mL) | 0.11  [0.11–0.11] | 0.11  [0.11–1.20] | 5.01  [4.33–6.36] | **0.037** |
| IL-10 (pg/mL) | 1.33  [0.81–4.68] | 11.02  [4.75–27.63] | 5.66  [2.97–21.37] | 0.481 |
| IL-17A (pg/mL) | 3.65  [3.30–4.89] | 4.69  [2.94–6.43] | 3.14  [2.94–3.34] | 0.734 |
| MCP-1 (pg/mL) | 4.10  [4.10–4.10] | 5.35  [4.73–24.82] | 56.63 *****  [35.42–57.72] | **0.035** |
| MIP-1α (pg/mL) | 0.08  [0.08–0.08] | 0.38 *****  [0.30–0.86] | 0.19  [0.14–0.62] | **0.040** |
| MIP-1β (pg/mL) | 0.60  [0.60–0.66] | 1.58  [0.95–2.14] | 0.95  [0.60–1.30] | 0.270 |
| MIP-2α (pg/mL) | 2.49 ± 1.50 | 5.07 ± 3.70 | 4.67 ± 4.65 | 0.630 |
| MMP-2 (ng/mL) | 415.85 ± 35.28 | 447.70 ± 46.30 | 369.87 ± 17.26 | 0.087 |
| MMP-3 (ng/mL) | 33.60  [28.45–35.25] | 43.80  [37.10–61.35] | 31.30  [25.50–37.10] | 0.135 |
| MMP-8 (ng/mL) | 124.60  [115.85–164.90] | 144.90  [124.05–203.90] | 116.70  [111.55–220.75] | 0.815 |
| proMMP-9 (ng/mL) | 12.37  [11.55–17.56] | 14.21  [11.03–26.80] | 7.72  [7.62–19.43] | 0.554 |
| MMP-12 (ng/mL) | 0.79 ± 0.07 | 0.44 ± 0.14 ***** | 0.59 ±0.21 | **0.042** |

* p<0.05 vs. APP23-Saline.

**Supplemental Table 7.** Demographic and clinical characteristics of the study cohort. ICH: Intracerebral hemorrhage.

|  | Lobar ICH  n=22 |
| --- | --- |
| Sex (female) | 13 (59.1%) |
| Age | 82.0 [70.0 ― 85.0] |
| MMP-12 (ng/mL) | 0.37 [0.25 ― 0.59] |
| HTA | 16 (72.7%) |
| Atrial fibrillation | 2 (9.1%) |
| Diabetes Mellitus | 3 (13.6%) |
| ICH volume (cm^3^) | 28.35 [14.65 ― 44.78] |
| Cholesterolemia | 7 (31.8%) |
| ApoEɛ2 | 4 (21.1%) |
| ApoEɛ4 | 4 (21.1%) |
| Creatinine (mg/dL) | 0.80 [0.72 ― 1.07] |
| Fibrinogen (g/L) | 4.13 ± 0.65 |
| Glucose (mg/dL) | 113.00 [95.0 ― 127.0] |
| Hemoglobin (g/dL) | 13.32 ± 1.94 |
| Anticoagulant treatment | 2 (8%) |
| International Normalized Ratio (INR) | 0.99 [0.93 ― 1.03] |
| Platelets (units/nL) | 214.73 ± 55.33 |
| Prothrombin time (s) | 12.10 [11.20 ― 13.30] |
| Partial thromboplastin time (s) | 27.30 [24.50 ― 30.70] |
| White blood cells (units/nL) | 8.83 ± 3.25 |

**Supplemental Table 8**. Associations between plasma MMP-12 levels and demographic and clinical variables from the ICH cohort represented by the p value obtained from the corresponding analysis.

| MMP-12 associations |  |
| --- | --- |
|  | **Lobar ICH**  **n=22** |
| Sex | 0.357 |
| Age | 0.762 |
| HTA | 0.590 |
| Atrial fibrillation | 0.364 |
| Diabetes Mellitus | 0.857 |
| ICH volume | ***0.059*** |
| ICH Shape | **0.011** |
| Cholesterolemia | 0.332 |
| ApoEɛ2 | 0.307 |
| ApoEɛ4 | 0.665 |
| Creatinine | 0.918 |
| Fibrinogen | 0.529 |
| Glucose | 0.649 |
| Hemoglobin | 0.231 |
| Anticoagulant treatment | 0.364 |
| International Normalized Ratio (INR) | 0.170 |
| Platelets | 0.587 |
| Prothrombin time | ***0.072*** |
| Partial thromboplastin time | 0.191 |
| White blood cells | 0.215 |

**Supplemental Table 9.** Backward linear regression with MMP-12 plasma levels in a lobar ICH cohort adjusting by sex, age and prothrombin time.

**A)**

|  | B [CI 95%] | p value |
| --- | --- | --- |
| ICH volume | 0.006 [0.000 ― 0.011] | **0.040** |
| Sex | – | – |
| Age | – | – |
| Prothrombin time | – | – |

**B)**

|  | B [CI 95%] | p value |
| --- | --- | --- |
| ICH Shape | 0.095 [0.007 ― 0.183] | **0.036** |
| Sex | – | – |
| Age | – | – |
| Prothrombin time | – | – |
